# Association of Inflammation and Endothelial Dysfunction with Coronary Microvascular Resistance in Patients with Cardiac Syndrome X

**DOI:** 10.5935/abc.20170149

**Published:** 2017-11

**Authors:** Ming Long, Zhibin Huang, Xiaodong Zhuang, Zena Huang, Yue Guo, Xinxue Liao, Chufan Luo

**Affiliations:** First Affiliated Hospital, Sun Yat-sen University, Guangzhou - China

**Keywords:** Acute Coronary Syndrome, Endothelium / physiopathology, Inflammation, Brachial Artery

## Abstract

**Background:**

Although a proportion of CSX patients have impaired brachial artery flow-mediated dilatation (FMD) in response to hyperemia, suggesting that endothelial dysfunction in these patients may be systemic and not just confined to the coronary circulation; the underlying mechanisms triggering endothelial dysfunction in these patients are still incompletely understood.

**Objectives:**

To assess the association of the index of Microcirculatory Resistance (IMR) with endothelial dysfunction and inflammation in patients with CSX.

**Methods:**

We studied 20 CSX patients and 20 age and gender-matched control subjects. Thermodilution-derived coronary flow reserve (CFR) and IMR were measured using a pressure-temperature sensor-tipped guidewire. Brachial artery FMD was measured using high-resolution, two-dimensional ultrasound images obtained with a Doppler ultrasound device (HDI-ATL 5000, USA) with a 5 MHz to 12 MHz linear-array transducer.

**Results:**

Compared with in control subjects, CFR was significantly lower (2.42 ± 0.78 vs. 3.59 ± 0.79, p < 0.001); IMR was higher (32.2 ± 8.0 vs. 19.5 ± 5.5, p < 0.001); the concentration of hs-CRP and FMD was higher (4.75 ± 1.62 vs. 2.75 ± 1.50; 5.24 ± 2.41 vs. 8.57 ± 2.46, p < 0.001) in CSX patients. The Duke treadmill score (DTS) was correlated positively to CFR and FMD (0.489 and 0.661, p < 0.001), it was negative to IMR and hsCRP (-0.761 and -0.087, p < 0.001) in CSX patients.

**Conclusions:**

The main finding in this study is that the DTS measured in patients with CSX was associated to hsCRP and FMD. Moreover, the independent effects of exercise tolerance can significantly impair FMD and hsCRP in CSX patients; especially it is particularly important to whom where FMD was associated negatively with IMR.

Although multiple pathophysiologic abnormalities have been reported in cardiac syndrome X (CSX), generalized endothelial dysfunction and inflammation are accepted as major pathophysiologic mechanisms.^1^ It has been proposed that because of endothelial dysfunction, reduced coronary vasodilatation and abnormal arterial constriction comes about in patients with CSX.^2^ It was also demonstrated that endothelium-dependent and independent dilatation is impaired in CSX. Masci PG et al^3^ have shown that a proportion of CSX patients have impaired brachial artery flow-mediated dilatation (FMD) in response to hyperemia suggesting that endothelial dysfunction in these patients may be systemic and not just confined to the coronary circulation. However, the underlying mechanisms triggering endothelial dysfunction in these patients are still incompletely understood. Studies have shown that impaired brachial artery FMD is significantly associated with elevated hs-CRP concentrations in patients with CSX^4^. These were an important role for inflammation in the modulation of coronary microvascular responses in patients with CSX.^5^

The Index of Microcirculatory Resistance (IMR) is measured at peak hyperemia, thereby eliminating the variability of resting vascular tone and hemodynamics. With recent technological advances, it is now possible to measure pressure while estimating coronary flow with a single pressure-temperature sensor-tipped coronary wire.^6^ Therefore, IMR is a readily available, quantitative and reproducible, wire-based method for invasively assessing coronary microvascular function independent of the epicardial artery in the cardiac catheterization laboratory.^7,8^

The aim of the present study was to assess the association of IMR with endothelial dysfunction and inflammation in patients with CSX.

## Methods

### Patient population and study protocol

The present prospective study was conducted in the Department of Cardiology, the First Affiliated Hospital of Sun Yat-sen University. We enrolled 20 CSX patients (CSX group) who fulfilled the diagnostic criteria for CSX in the present study as follow:^5^ (1) a typical history of exertional angina; (2) a positive exercise treadmill test; and (3) angiographically normal epicardial coronary arteries without minimal irregularities.

This study was our series of research on coronary microvascular dysfunction, and for the first time, we directly demonstrated the relationship of inflammation and endothelial dysfunction in increased microvascular resistance in CSX patients in the small size sample of our previous study.^9^

The control group consisted of 18 age- and gender-matched subjects who were referred for diagnostic coronary angiography due to atypical chest discomfort, with a negative exercise treadmill test and completely normal coronary arteries at angiography.

Subjects with any of the following clinical conditions were excluded from this study: other specific forms of cardiac disease (for example, variant angina, cardiomyopathies, and valvular or congenital heart disease), any regional wall motion abnormalities at resting echocardiogram, ejection fraction less than 50%, atrial fibrillation or left bundle branch block on ECG, uncontrolled hypertension (> 160/100 mmHg) or diabetes mellitus (fasting plasma glucose > 7.0 mmol/L and/or postprandial glucose > 11.0 mmol/L), systemic disorders, and liver or renal insufficiency.

The hospital Ethics Committee approved the study protocol, and each subject gave written informed consent to the study.

### Coronary angiography

In all study participants, selective coronary angiography was performed using standard Judkins technique. Coronary arteries were visualized in left and right oblique planes with cranial and caudal angulations. Injection of contrast medium (Iopromide, Ultravist-370; Schering AG, Berlin, Germany) was carried out by an automatic injector, at a speed of 5 ml/s for left coronary artery and 3 ml/s for right coronary artery.

All anti-angina and anti-ischemic medications, except sublingual nitroglycerin, were withheld for at least one week before the examination. All coronary angiograms were analyzed by two experienced independent investigators and only angiograms with visually smooth contours with no wall irregularities were accepted as normal.

### Intracoronary thermodilution measurements

After coronary angiography, a coronary pressure wire (PressureWire-4, Radi Medical Systems, Wilmington, Mass.) was calibrated outside the body, equalized to the guiding catheter pressure with the sensor positioned at the ostium of the guiding catheter, and then advanced it until the wire sensor was located in the distal third of the left anterior descending coronary artery, with the transducer distance at 7~10 cm from the guide tip.

With commercially available software (Radi Medical Systems), the shaft of the pressure wire can act as a proximal thermistor by detecting changes in temperature-dependent electrical resistance. The sensor near the tip of the wire simultaneously measures pressure and temperature and can thereby act as a distal thermistor. The transit time of room-temperature saline injected down a coronary artery can be determined using a thermodilution technique.^5^ Three injections of saline (3 mL, room temperature) were administered into the coronary artery, and the baseline mean transit time (bTmn) was measured. Intravenous adenosine (140 ug/kg/min) was then administered to induce steady-state maximal hyperemia, then three more injections of saline (3 mL, room temperature) were given, and the hyperemic mean transit time (hTmn) was measured. Simultaneous measurements of mean aortic pressure (P*a*, by guiding catheter) and mean distal coronary pressure (P*d*, by pressure wire) were also made in the resting and maximal hyperemic states. CFR was calculated as bTmn divided by hTmn. IMR was calculated as the distal coronary pressure at maximal hyperemia divided by the inverse of hTmn. Fractional flow reserve (FFR) was calculated by the ratio of P*d*/P*a* at maximal hyperemia.

### Measurement of FMD in the brachial artery

Brachial artery FMD was measured using a standardized technique.^10^ All subjects were studied in the morning, having abstained from alcohol, caffeine and food for 12 h before the test. The diameter of the artery was measured using high-resolution, two-dimensional ultrasound images obtained with a Doppler ultrasound device (HDI-ATL 5000, USA) with a 5 MHz to 12 MHz linear-array transducer. The right brachial artery was scanned over a longitudinal section, approximately 3 cm to 5 cm above the right elbow. Depth and gain settings were optimized to identify the lumen-to-vessel wall interface. An ECG monitor integrated with the ultrasound machine was also applied. When an optimal image of the brachial artery was obtained, the surface of the skin was marked, and the arm was maintained in the same position throughout the study. Following the measurement of baseline brachial artery diameter, a pneumatic tourniquet placed around the forearm distal to the target artery was inflated to a pressure of 250 mmHg, and inflation was held for 5 min. Reactive hyperemia in the brachial artery was then induced by rapid cuff deflation. A second scan was performed continuously for 60 s before and 120 s after cuff deflation. The ultrasound images were recorded using digital video. The diameter of the brachial artery was measured from the anterior to the posterior interface between the media and adventitia at a fixed distance. The mean diameter was calculated from four cardiac cycles synchronized using the R-wave peaks on the ECG. All measurements were recorded at end-diastole to avoid possible errors resulting from variable arterial compliance. Maximal vasodilation was observed 60 s after cuff release. FMD was calculated as the percentage change in artery diameter from baseline to reactive hyperemia. All of the ultrasonographic assessments were performed by the same blinded radiologist.

### Serum high-sensitive C-reactive protein measurements

Blood samples were immediately centrifuged at 2000 g for 10 min, and serum was stored at −80ºC for the subsequent analysis of biochemical markers. The level of hsCRP was measured at 550 nm by a particle enhanced immunoturbidimetric assay (Orion Diagnostic, USA) on a Hitachi 7170A (Hitachi, Japan) analyzer. The lower limit of detection for the method is 0.125 mg/L. The interassay coefficient of variations were 0.14% and 2.1% at mean values of 8.20 mg/L and 0.31 mg/L, respectively; the intra-assay coefficients of variation were 0.13% and 2.3% at mean values of 8.21 mg/L and 0.30 mg/L, respectively. An hs-CRP concentration > 3 mg/L was considered abnormal as suggested previously.^11^

### Statistical analysis

The statistical analysis was performed using SPSS 13.0 software. Data were expressed as mean ± SD unless otherwise specified. Categorical variables were presented using the absolute number and its proportion. Continuous variables between groups were compared by paired Student’s *t* test. Proportions were compared by the Fisher exact test when the expected frequency was < 5, otherwise the chi-square test was applied. If the data followed a normal distribution, two-sample independent Student’s T test was used; otherwise, two-sample Wilcoxon Rank Sum Test was used. Pearson’s correlation analysis was used to test univariate relations. Significance was considered to be achieved for two-tailed p values < 0.05.

## Results

### Study population and clinical demographics in two group of patients

Patients’ clinical demographics and characteristics for the use of medications in both groups are documented in Table 1. The two groups were similar for age, gender, height, weight, cardiovascular risk factors, blood pressure, heart rate, and left ventricular function, and there were no differences in characteristics for the use of medications between these two groups.

**Table 1:** Clinical Demographics in cardiac syndrome X patients and controls

Variable	CSX group (n = 20)	Control group (n = 20)	p Value*
Age, y	53.6 ± 9.8	54.7 ± 10.0	0.727
Male gender, n (%)	5 (25.0%)	6 (30.0%)	0.709
Height, cm	162.0 ± 8.0	161.9 ± 7.5	0.968
Weight, kg	63.2 ± 7.6	62.3 ± 8.3	0.730
**Risk factors, n (%)**			
Systemic hypertension	10 (50.0%)	7 (35.0%)	0.312
Diabetes mellitus	5 (25.0%)	4 (20.0%)	0.690
Hypercholesterolemia	4 (20.0%)	6 (30.0%)	0.441
Current smokers	2 (10.0%)	3 (15.0%)	0.614
Family history of coronary disease	3 (15.0%)	2 (10.0%)	0.614
Systolic blood pressure, mmHg	127.8 ± 12.2	125.9 ± 12.6	0.622
Diastolic blood pressure, mmHg	73.7 ± 10.2	73.8 ± 6.9	0.971
Rest heart rate, beats/min	70.5 ± 7.5	68.2 ± 8.7	0.376
Left ventricular ejection fraction, %	58.9 ± 7.6	60.3 ± 6.8	0.555
**Use of medication, n (%)**			
Aspirin	16 (80.0%)	16 (80.0%)	1.000
Statins	12 (60.0%)	9 (45.0%)	0.317
Beta-blockers	11 (55.0%)	7 (35.0%)	0.180
ACE inhibitors and/or ARB	4 (20.0%)	6 (30.0%)	0.441

Values are given as number of patients (%) or mean ± SD. ACE: angiotensin-converting enzyme; ARB: angiotensin II receptor blocker;

(*) Mann-Whitney test.

### Comparisons of CFR, IMR and FFR between groups

No significant difference was present in exercise duration between groups. Thermodilution-derived IMR could be easily measured in all studied cases. CFR and IMR were significantly lower in CSX group than in Control group, and the FFR values were not different in two groups. The mean values for CFR and IMR was 2.42 ± 0.78 vs 3.59 ± 0.79 and 32.2 ± 8.0 vs 19.5 ± 5.5, whereas FFR (0.95 ± 0.02 and 0.94 ± 0.03) showed no differences (p > 0.05 for all comparisons) (Table 2).

**Table 2:** Comparisons of coronary physiology and exercise test results in cardiac syndrome X patients and controls

Variable	CSX group (n = 20)	Control group (n = 20)	p Value*
CFR	2.42 ± 0.78	3.59 ± 0.79	< 0.001
IMR	32.2 ± 8.0	19.5 ± 5.5	< 0.001
FFR	0.95 ± 0.02	0.94 ± 0.03	0.470
Exercise duration (minutes)	7.60 ± 2.30	9.20 ± 2.86	0.059
**Net exercise-induced**			
ST-segment deviation (millimeters)	1.85 ± 0.75	0.25 ± 0.25	< 0.001
Treadmill angina index	0.45 ± 0.69	0.00 ± 0.00	0.006
Double product (mmHg.bpm)	26051 ± 6177	27366 ± 5247	0.472
DTS	-2.2 ± 7.7	8.1 ± 3.3	< 0.001
Serum hsCRP, mg/L	4.75 ± 1.62	2.75 ± 1.50	< 0.001
Brachial FMD, %	5.24 ± 2.41	8.57 ± 2.46	< 0.001

Values are given as mean ± SD. CFR: coronary flow reserve; IMR: index of microvascular resistance; FFR: fractional flow reserve; DTS: Duke treadmill score; bpm: beats per minute; FMD: flow-mediated dilation;

(*) Mann-Whitney test.

### Relationships between hs-CRP, FMD, IMR and DTS

In CSX patients, the DTS was correlated negatively to IMR (r = -0.761, p < 0.001) and positively to FMD (r = -0.661, p = 0.002) with CFR (r = 0.489, p = 0.029), while evaluating correlations of myocardial ischemic threshold with IMR and CFR, we found that exercise duration was correlated positively to FMD (r = 0.448, p = 0.048) with CFR (r = 0.599; p = 0.005) and negatively to IMR (r=−0.604; p = 0.005), however, the DTS and exercise duration was not relevant with hsCRP (r = -0.087, p = 0.716 and r = 0.016, p = 0.948) and FFR (r = -0.309, p = 0.761and r = -0.091, p = 0.703). Importantly, the FMD was associated negatively with IMR (r = -0.869, p < 0.001). Interestedly, double product was significantly correlated to hsCRP (r = -0.502; p = 0.024) and not significantly correlated to FMD (r = 0.328, p = 0.158), CFR (r = 0.429, p = 0.059), IMR (r = -0.399, p = 0.082), and FFR (r = 0.015, p = 0.951) (Table 3).

**Table 3:** Pearson correlation between hs-CRP, FMD, IMR and DTS in cardiac syndrome X patients and controls group

Variable	CSX group (correlation coefficient)			Control group (correlation coefficient)		
	DTS (p value)	exercise duration (p value)	double product (p value)	DTS (p value)	exercise duration (p value)	double product (p value)
CFR	0.489* (0.029)	0.599* (0.005)	0.429 (0.059)	0.241 (0.307)	-0.126 (0.598)	0.229 (0.332)
IMR	-0.761* (0.000)	-0.604* (0.005)	-0.399 (0.082)	0.156 (0.511)	0.053 (0.823)	-0.258 (0.272)
FFR	-0.073 (0.761)	0.091 (0.703)	0.015 (0.951)	0.143 (0.548)	0.252 (0.284)	0.293 (0.210)
hsCRP, mg/L	-0.087 (0.716)	0.016 (0.948)	-0.502* (0.024)	-0.006 (0.980)	-0.072 (0.763)	-0.430 (0.058)
FMD, %	0.661* (0.002)	0.448* (0.048)	0.328 (0.158)	-0.254 (0.279)	-0.092 (0.701)	0.036 (0.880)

CFR: coronary flow reserve; IMR: index of microvascular resistance; FFR: fractional flow reserve; DTS: Duke treadmill score; bpm: beats per minute; FMD: flow-mediated dilation; (*p < 0.05, Statistical test performed: linear regression analysis and Pearson's correlation coefficient).

In control group, the DTS, exercise duration and double product was all not correlated to hsCRP, FMD, CFR, IMR, and FFR.

## Discussion

In our past study, we firstly found that coronary microvascular dysfunction in patients with CSX was represented by the increased IMR and the impaired CFR. For the further study, the main finding in this study is that the DTS measured in patients with CSX was associated of hsCRP and FMD. An interesting secondary finding is that double product was significantly correlated to hsCRP. These findings reinforce the importance of inflammation and endothelial dysfunction in the microvascular dysfunction of CSX patients. They also highlight the independent effects of exercise tolerance can significantly impair FMD and associated of hsCRP in CSX patients. Thirdly, it is particularly important to that the FMD was associated negatively with IMR.

IMR is a quantitative, reproducible index that is independent of epicardial coronary disease and specific for the microcirculation. Our previous studies suggested that IMR might be a more superior and reliable index reflecting coronary microcirculatory function. CSX patients are often strongly symptomatic and difficult to manage, which affects microvascular systems and increases vascular tonus. In our past study, coronary microvascular dysfunction in CSX patients was associated with increased IMR, we further documented that the FMD was associated negatively with IMR. It has been suggested that coronary microvascular dysfunction is caused by showed a correlation between coronary microvascular endothelial dysfunction and peripheral vascular dilatation. Our findings also agree with data from Tondi P et al.,^12^ who reported FMD in patients who had cardiac syndrome X.

Several studies have demonstrated that endothelial dysfunction and inflammation can be pathophysiological mechanisms of CSX patients, and our results were completely consistent with these opinions.^13,14^ Traditional cardiovascular risk factors, insulin resistance,^15^ and estrogen deficiency^16^ have been reported to be highly prevalent in CSX patients and probably contribute to microcirculatory dysfunction in these patients. Moreover, Ong et al.^4^ confirmed a significant correlation between obesity and reduced flow mediated dilatation as well as with hs-CRP concentrations, and they suggested that obesity contributes to low-grade inflammation and impaired FMD in CSX patients. Further studies confirmed that impairment balance between tPA, PAI-1 endothelin-1 and NO, increased sympathetic tonus, abnormal coronary flow reserve, and microvascular spasm were possibly one of the mechanisms of CSX.^17-22^ The level of C reactive protein in patients with chest pain and normal coronary arteries correlates with the frequency and duration of chest pain and the extent of ST-segment depression on exercise testing and ambulatory monitoring.^23^ Verma et al.^24^ reported that recombinant human CRP directly inhibits endothelial progenitor cells differentiation, survival and function at concentrations known to predict adverse vascular outcomes. Therefore, the enhanced extent of inflammation observed in patients with CSX may reduce endothelial progenitor cells levels and function in blood circulation, resulting in attenuated repair capacity of vasculature. Our data supported these findings that hsCRP was significantly correlated to DTS and double product in CSX patients, and it was suggested that exercise tolerance can significantly associated of hsCRP in CSX patients. This suggests that the inflammatory damaged vascular endothelial function, further affecting the cardiac microvascular reserve function, influenced the exercise tolerance in CSX patients.

FMD is a noninvasive index of endothelial function and vascular health in humans. During the past decades, a large body of evidence accumulated has indicated that an impaired FMD is detectable in CSX patients, suggesting a generalized abnormality in vascular function.^11^ Our data also shows the same result that FMD was associated with IMD and it reflected the microvascular function in CSX patients. Masci et al.,^3^ showed that these patients with lower FMD responses had a higher probability of having transient myocardial perfusion defects on thallium-201 single-photon emission computed tomographic. Furthermore, Lekakis et al.^25^ suggested that CSX patients had significantly lower brachial artery FMD in response to hyperemia than healthy controls. In addition, it has been demonstrated that coronary microvascular dysfunction as well as peripheral artery endothelial dysfunction in CSX can be associated with elevated CRP concentrations. Teragawa et al. 26 showed, for example, that impaired microvascular coronary responses to intracoronary acetylcholine were significantly more common in patients with chest pain, normal coronary arteriograms and elevated CRP levels compared to patients with normal CRP concentrations.

### Study limitations

This is a prospective, observational study designed to demonstrate invasively coronary microvascular dysfunction in CSX patients. The primary limitation was the comparatively small number of patients, but the diagnostic criteria we adopted were well-characterized, including only patients with completely normal coronary angiograms, effort-induced angina pectoris, and positive exercise stress test, which resulted in a relatively low incidence of CSX. Second, we did not exclude subjects with traditional cardiovascular risk factors, such as hypertension, diabetes mellitus, hypercholesterolemia or smoking, which can also influence vascular function and lead to CMD. However, the CSX patients were strictly matched to a control group, with no differences between groups in clinical demographics and cardiovascular risk factors, which gives strength to our findings.

## Conclusion

The main finding in this study is that the DTS measured in patients with CSX was associated of hsCRP and FMD. Moreover, the independent effects of exercise tolerance can significantly impair FMD and be associated with hsCRP in CSX patients, especially, it is particularly important to those where FMD was associated negatively with IMR.
